# Increased sinusoidal flow is not the primary stimulus to liver regeneration

**DOI:** 10.1186/1476-5926-9-2

**Published:** 2010-01-20

**Authors:** Kim E Mortensen, Lene N Conley, Ingvild Nygaard, Peter Sorenesen, Elin Mortensen, Christian Bendixen, Arthur Revhaug

**Affiliations:** 1 Surgical Research Laboratory, Institute of Clinical Medicine, University of Tromsoe, Tromsoe, Norway; 2 Faculty of Agricultural Sciences, Department of Genetics and Biotechnology, University of Aarhus, Aarhus, Denmark; 3 Department of Pathology, University Hospital of Northern-Norway, Tromsoe, Norway; 4 Department of Gastrointestinal Surgery, University Hospital of North-Norway, Tromsoe, Norway

## Abstract

**Background:**

Hemodynamic changes in the liver remnant following partial hepatectomy (PHx) have been suggested to be a primary stimulus in triggering liver regeneration. We hypothesized that it is the increased sinusoidal flow per se and hence the shear-stress stimulus on the endothelial surface within the liver remnant which is the main stimulus to regeneration. In order to test this hypothesis we wanted to increase the sinusoidal flow without performing a concomitant liver resection. Accordingly, we constructed an aorto-portal shunt to the left portal vein branch creating a standardized four-fold increase in flow to segments II, III and IV. The impact of this manipulation was studied in both an acute model (6 animals, 9 hours) using a global porcine cDNA microarray chip and in a chronic model observing weight and histological changes (7 animals, 3 weeks).

**Results:**

Gene expression profiling from the shunted segments does not suggest that increased sinusoidal flow per se results in activation of genes promoting mitosis. Hyperperfusion over three weeks results in the whole liver gaining a supranormal weight of 3.9% of the total body weight (versus the normal 2.5%). Contrary to our hypothesis, the weight gain was observed on the non-shunted side without an increase in sinusoidal flow.

**Conclusions:**

An isolated increase in sinusoidal flow does not have the same genetic, microscopic or macroscopic impact on the liver as that seen in the liver remnant after partial hepatectomy, indicating that increased sinusoidal flow may not be a sufficient stimulus in itself for the initiation of liver regeneration.

## Background

Since Higgins and Anderson formalized the study of liver regeneration in 1931 [1] most studies have been conducted in a model of 70% partial hepatectomy (PHx) in rodents. Following PHx, several pro-mitotic (IL-1, IL-6, EGF, HGF, TNFα) and pro-apoptotic factors (TGFβ, Fas ligand) are known to be important substances regulating the initiation, propagation and termination of liver regeneration [2-5]. Many of these blood borne factors are detectable several hours after PHx [6-8], and constitute the basis for the well established "humoral theory" of liver regeneration.

However, later studies have shown that liver regeneration commences already 15 minutes after PHx (via the detection of c-fos mRNA) suggesting more immediate triggering events [9]. Several studies indicate that the increased portal pressure and flow per gram remaining liver tissue and hence sinusoidal shear stress that occurs immediately following PHx may be a primary stimulus to regeneration [7,10,11]. Endothelial shear stress results in the production of Nitric Oxide (NO) in the liver [12,13] and several studies have illustrated that liver regeneration is inhibited by administration of the NO synthase antagonist *N*G-nitro-L-arginine methyl ester (L-NAME) and restored by the NO donor 3-morpholinosydnonimine-1 (SIN-1) [9,14,15]. Consequently, a "flow theory" on liver regeneration has emerged. Yet, to the best of our knowledge, no study to date has been conducted where shear stress as the sole stimulus has been quantified in-vivo together with the local hepatic NO production. Thus, the link between shear stress, NO production and the triggering of regeneration is still unclear.

More recent studies on the genetic regulation of the regeneration cascade have employed microarray analysis [16-20] in rodent models of PHx using liver specific chips and collectively describe gene expression profiles in the regenerating liver over a time span of one minute to one week after resection. Using a novel global porcine cDNA chip, we recently demonstrated that the immediate genetic regenerative response in the porcine liver remnant varies according to the volume of resection and rise in portal venous pressure in the pig. We also found differentially expressed genes in the liver remnant after a 75% PHx to have functions primarily related to apoptosis, nitric oxide metabolism and oxidative stress, whereas differentially expressed genes in the liver remnant after a 62% PHx primarily promoted cell cycle progression [21]. In our opinion, this partially corroborates the "flow theory" of liver regeneration because the genetic response is influenced by changes in the portal pressure increase and differences in flow per gram liver tissue in the respective remnants.

However, the hemodynamic changes in the liver remnant resulting from PHx results not only in increased flow and shear stress in the remaining sinusoids, but also increased delivery of hepatotrophic factors to the replicating hepatocytes. Therefore, to distinguish the effects of these two potentially different stimuli (increased sinusoidal flow/shear-stress versus increased delivery of hepatotrophic factors), and further scrutinize the potential effects of increased sinusoidal flow, we hypothesized in the present study that, according to the "flow theory" of liver regeneration, it is the increased sinusoidal flow in itself, which is the primary stimulus to liver regeneration. Consequently, selectively increasing the flow to segments II, III and IV should, lead to similar gene expression profiles as those seen shortly after PHx, and over time, lead to hyperplasia/hypertrophy of these segments.

To create an isolated, regional increased sinusoidal flow in-vivo without simultaneous liver resection, we manipulated the hepatic blood supply by creating an aorto-portal shunt to the left portal vein branch, thereby selectively increasing the flow to segments II, III and IV to a similar flow rate (per gram liver) as that seen after a 75% PHx [21]. This was done in a set of acute experiments, shunting these segments over a period of 6 hours, analyzing cell cycle regulatory genes and also in a separate set of chronic experiments over three weeks, measuring segmental liver weight and histological changes.

The results of the present study show that an isolated increase in sinusoidal flow does not have the same impact on the liver as that seen in the liver remnant after partial hepatectomy, indicating that increased sinusoidal flow may not be a the primary stimulus for the initiation of liver regeneration

## Methods

### Animal preparation

**Figure 1:**
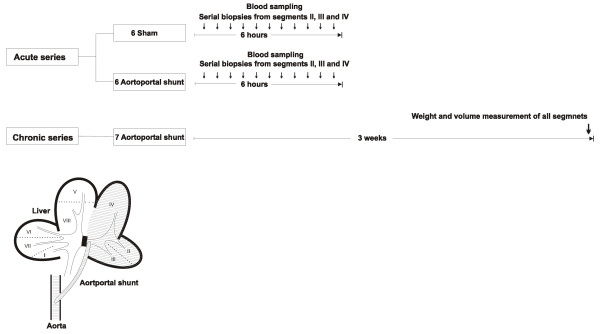
**Experimental setup**. In the acute series, flow and pressure in all vascular structures to the liver were recorded continuously for the whole experiment. In the chronic series, flow in the aortoportal shunt was recorded upon establishment and after three weeks upon relaparatomy.

Fig. 1 displays the experimental setup. All experiments were conducted in compliance with the institutional animal care guidelines and the National Institute of Health's *Guide for the Care and Use of Laboratory Animals *[DHHS Publication No. (NIH) 85-23, Revised 1985]. A total of nineteen pigs were used (*Sus scrofa domesticus*), aged approximately 3 months; twelve in the acute experiments, with an average weight of 33.5 kg (± 2 kg) and seven in the chronic experiments, with an average weight of 31.0 kg (± 2 kg). In the acute series, we followed the same anesthesia protocol as previously described [21]. In the chronic series, anesthesia for the surgical intervention was maintained with isoflurane 1.5-2% mixed with 55% oxygen. Respiratory rate was adjusted to achieve an Et CO2 between 3.5 and 6 KPa. Mean alveolar concentration of isoflurane was maintained at 1.3 using a Capnomac (Nycomed Jean Mette). Analgesia was induced and maintained with fentanyl 0.01 mg/kg. Before surgery, all animals received a single i.m. shot of antibiotic prophylaxis (Enrofloxacin, 2.5 mg/kg).

### Catheters

In the acute series, a 16G central venous catheter (CVK, Secalon^® ^T) was placed in the left external jugular vein for administration of anesthesia and infusions. A 5 French Swan-Ganz catheter (Edwards Lifesciences™) was floated via the right external jugular vein to the pulmonary artery for cardiac output (CO) measurements. A 16G CVK (Secalon^® ^T) was placed in the left femoral artery for continuous arterial blood pressure monitoring. A 7 French 110 cm angiographic catheter (Cordis^®^, Johnson&Johnson™) was placed in the right hepatic vein draining segments V, VI, VII and VIII via the right internal jugular vein for blood pressure monitoring and blood sampling. A 5 French Swan-Ganz catheter (Edwards Lifesciences™) was placed in the hepatic vein draining segments II and III by direct transhepatic placement for pressure monitoring and blood sampling. Inflation of the balloon allowed wedged hepatic pressure measurement. A pediatric CVK (Arrow^® ^International) was placed in the portal vein for blood pressure monitoring and blood sampling.

No catheters were placed in the pigs in the chronic series, as the main objective here was to anastomose the shunt from the aorta to the left portal vein branch with minimal damage to the hepatic hilus.

### Measurements

#### Acute series

Calibrated transducers (Transact 3™, Abbott Critical Care Systems, Chicago, IL, USA) were used for continuous pressure registration and signals were stored electronically (Macintosh Quadra 950, Apple Computers, CA, USA). Perivascular ultrasonic flow probes (CardioMed Systems, Medistim A/S, Oslo, Norway) were placed around the portal vein, right hepatic artery, left hepatic artery and around the aortoportal shunt. Cardiac output was measured by thermo dilution (Vigilance™ Volumetrics, Edwards Lifesciences™). Measurements were made in triplicate and averaged. The heart rate was monitored with an electrocardiogram (ECG).

#### Chronic series

The heart rate was monitored with an ECG. Flow in the aortoportal shunt was measured using an 8 mm perivascular ultrasonic flow probe (CardioMed Systems, Medistim A/S, Oslo, Norway).

### Surgery

#### Acute series

After a midline laparotomy and placement of all catheters and flow probes as described above, we isolated and recorded the flow in the left portal vein branch (LPVB). When the activated clotting time (ACT) was above 250 seconds, a 5 mm Propaten Gore-Tex™ graft was anastomosed end-to-side from the aorta (between truncus coeliacus (TC) and the superior mesenteric artery (SMA)) to the LPVB. The LPVB was then ligated proximal to the bifurcation to prevent backflow to the main portal vein trunk (MPVT). The opening of the shunt was regarded as time = 0 and noted. Flow in the shunt was standardized in each experiment to 1000 mL/minute by gradual shunt constriction using a ligature and a perivascular flow probe (Fig. 1). Sham surgery consisted of all the steps above except for the establishment of the aortoportal shunt.

#### Chronic series

After a midline laparotomy, a similar shunt was placed from the aorta to the LPVB once the animal had received 5000 IE heparin i.v. We used an interposed aorta graft from a donor pig (as the Gore-Tex grafts™ tended to become occluded). The LPVB was ligated proximal to the portal bifurcation to prevent backflow to the MPVT. Flow was standardized (by concentric constriction with a ligature) to 1000 mL/minute. Upon relaparatomy three weeks later, the shunt was isolated and flow measured. The flow in the MPVT (now supplying the right liver only) was recorded.

### Sampling

In the acute series, sequential biopsies were taken from the shunted segments II, III and IV at time points 1, 5, 10, 30, 60, 90 minutes and 2, 3, 4 and 6 hours after shunt opening (t = 0). The sampling time points were the same as in a previous study of liver regeneration after PHx [21] using the same microarray platform allowing the direct comparison of gene expression profiles found in the present experiments with the former. Biopsies were placed immediately in RNAlater (Ambion^®^).

Blood extraction was performed before biopsy sampling. Samples were taken from the portal vein, femoral artery, and hepatic vein draining both sides of the liver. Aspartate aminotransferase (ASAT), alanine aminotransferase (ALAT), glutamyl transpeptidase (GT), glucose, bilirubin (Bil) and alkaline phosphatase (ALP) levels were quantified by calorimetric, ultraviolet-photometric, and HPLC analysis (Roche, PerkinElmer).

For cytokine analysis, a multiplex kit was developed including four different cytokines; TNF-α, IL-1α, IL-6 and IL-10. Serum samples was analyzed in duplicates using the Luminex 200™ with the Bioplex manager software (BioRad, Hercules, CA) [22].

In the sham series, liver biopsies were taken from segments II, III and IV and blood was sampled from the same locations at the same time points as in the shunted animals.

In the chronic series, only peroperative arterial blood gas samples were taken (directly from the aorta) to monitor respiratory status.

### Histological assessment

To evaluate the long-term (3 weeks) effects of arterial hyperperfusion on the liver parenchyma we took biopsies from both the shunted and the portally perfused sides of the liver before and after shunting. Specimens were fixed in buffered formalin, paraffin embedded, and stained with hematoxylin-eosin (HE) to evaluate tissue architecture. To evaluate proliferative activity, sections were stained with Ki67 and phosphohistone H3. The proliferative index was estimated by counting the number of Ki67 positive cells relative to the number of non-stained hepatocytes per liver lobuli. Connective tissue distribution was studied using reticulin staining. An independent pathologist (EM) reviewed the sections in a blinded manner.

### Microarray analysis

Two-color microarray analyses of the samples from the acute series were conducted to identify genes being significantly differentially expressed between the different time-points. The microarray experiment was conducted as a common reference design using liver total-RNA purified from an unrelated animal as the reference. Total-RNA was extracted and aminoallyl-cDNA (aa-cDNA) was synthesized from 20 μg of total-RNA. The reference samples were labeled with Alexa 488 and individual samples were labelled with Alexa 594. The samples were hybridized to the pig array DIAS_PIG_55K3, which consist of 26,879 PCR products amplified from unique cDNA clones. Following hybridization, washing and drying, the slides were scanned and the median intensities were computed. Statistical analysis was carried out in the R computing environment using the Bioconductor package Limma. The log2-transformed ratios of Alexa-594 to Alexa-488 were normalized within-slide using the loess function and were analyzed to identify genes being significantly differentially expressed by time within treatment as well as between treatments. Time contrasts were formed referring to the sample taken at time point 1 min. Furthermore, multiple testing across contrasts and genes was conducted. The false discovery rate was controlled using the method of Benjamini and Hochberg [23] as implemented in Limma. The genes were further analyzed by utilizing information from Online Mendelian Inheritance in Man (OMIM, [24]) to group the genes by function. More detailed descriptions of the microarray experiments are available at the NCBIs Gene Expression Omnibus [25,26] through the GEO series accession number GSE13683.

### Statistical analysis

Substrate flux across the liver remnant was analyzed using linear mixed models in SPSS 15, testing time (T), and group*time (GT) interaction. P values ≤ 0.05 were considered significant. Analysis of differences in hemodynamic changes between the shunt- and sham groups was analyzed using scale-space analysis of time series [27]. Comparison of group differences at specific time points was done using a two-tailed Student's t-test with the Bonferroni correction for multiple measurements. Results are expressed as mean values ± SD.

## Results

### Hemodynamics of the acute series (Additional file 1: Table S1)

Upon opening the shunt, the mean arterial pressure (MAP) decreased from 90.3 to 70.3 mmHg (*p *= 0.01). The systemic vascular resistance (SVR) fell from 16.5 to 11.2 mmHg min/mL (*p *= 0.002). A reciprocal increase in heart rate from 100 to 150 beats per minute (*p *< 0.05) and a sustained increase in cardiac output (CO) from 5.01 to 6.65 mL/minute was observed (not significant due to large standard deviation). This was in contrast to the sham animals, where these parameters remained unchanged throughout the same time period.

The flow in the LPVB increased from the normal average of 221 ml/minute of portal blood flow to an average of 1050 ml/minute of arterial blood flow as a result of the aortoportal shunting. This increased the flow/gram liver in the shunted side by a factor of 4.7 from 0.61 mL/minute/gram to 2.89 mL/minute/gram (*p *< 0.001). The flow in the right portal vein branch (RPVB) decreased slightly from 647 mL to 636 mL after ligating the LPVB. Hereafter, the flow fell gradually throughout the experiment, the flow becoming increasingly lower over time compared to the sham group (*p *= 0.01). No significant change in flow per gram liver in the portally perfused segments was observed (1.57 mL/minute/gram to 1.53 mL/minute/gram).

Conversely, the portal venous pressure (PVP) (in the MPVT) increased in the shunt group from an average of 6.22 to 8.55 mmHg (after ligation of the LPVB) whilst the PVP decreased in the sham group from an average of 6 to 5 mmHg, the pressure change trends being significantly different in the two groups (*p *< 0.05).

Upon opening the shunt, the flow fell abruptly in the left hepatic artery from 169 to 122 mL/min and continued to fall significantly throughout the experiment (*p *= 0.023). The flow in the right hepatic artery also decreased abruptly from 85 to 46 mL/min upon opening the shunt and fell in a similar manner over time (*p *= 0.022).

The free hepatic venous pressure remained unchanged in both right and left hepatic veins in both shunt and sham groups. However, the wedged pressure in the left hepatic vein in the shunt group increased significantly from 2.33 to 8 mmHg over six hours, in contrast to the sham group where the pressure remained unchanged (group*time interaction, *p *= 0.003).

### Hemodynamics of the chronic series (Additional file 1: Table S1)

Shunt: the average flow in the aortoportal shunt at opening of the shunt, t = 0, was 1007 mL/minute. Upon relaparotomy (t = 3 weeks), this had increased to1496 mL/minute (*p *= 0.004). However, the weight of the segments hyperperfused (segments II, III and IV) also increased from 341.5 grams (calculated by using data from a weight matched group of 6 pigs) to 633.9 grams (*p *= 0.0001), thus the flow per gram liver decreased from 2.97 to 2.38 mL/minute/gram (*p *= 0.045).

Portal flow: to avoid postoperative morbidity due to damage and following leakage of the lymphatics in the liver hilus, we did not expose the main portal vein trunk at t = 0 in the chronic series. The average flow in the main portal trunk at t = 0 was therefore calculated by using data from a weight matched group of 12 pigs where the average flow in the main portal vein was 850 mL/minute. By adjusting the flow to segments I, V, VI, VII and VIII, according to the weight that these segments comprised, the flow was calculated to be 459 mL/minute (± 74) to these segments. At relaparatomy (t = 3 weeks) the flow in the portal vein (now supplying only the right liver, segments I, V, VI, VII and VIII) was 1120 mL/minute. Accordingly, the flow to these segments had increased significantly (*p *= 0.008). However, due to the weight increase of these segments over three weeks, the flow per gram liver actually decreased from 2.07 to 1.08 mL/minute/gram (*p *< 0.0001).

### Macroscopic changes in the chronic series

**Figure 2:**
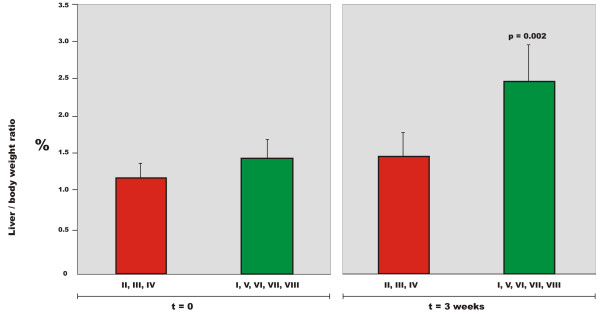
**Liver/body weight ratio (%) by segments before and after 3 weeks of aortoportal shunting of segments II, III and IV**. The total liver weight increases over three weeks, the increase occurring in the non-shunted segments (I, V, VI, VII and VIII).

Over a period of three weeks the pigs gained weight from 30.9 to 41.9 Kg (*p *= 0.0002). The total liver weight of six weight-matched pigs was 754 grams (± 107) at t = 0. After three weeks, the total liver weight in the shunted pigs had increased to 1667 grams (± 223) (*p *= < 0.0001). By calculating the liver weight/body weight percentage we get an increase from 2.74% at t = 0 to 3.99% at t = 3 weeks (*p *= 0.004). The weight of segments I, V, VI, VII and VIII in the weight-matched pigs at t = 0 was 412.8 grams (± 71.5). The weight of these segments at t = 3 weeks in the shunted animals was 1034.5 grams (± 166.5). The weight of segments II, III and IV at t = 0 was 341.6 (± 36.9). The weight of these segments at t = 3 weeks was 633.3 grams (± 109.2). Calculating the liver weight/body weight ratio by segments we found an increase in % for segments I, V, VI, VII and VIII from 1.49 to 2.47% (*p *= 0.002) and for segments II, III and IV from 1.24 to 1.52% (not significant) (Table S1, Additional file 1 and Fig. 2).

**Figure 3:**
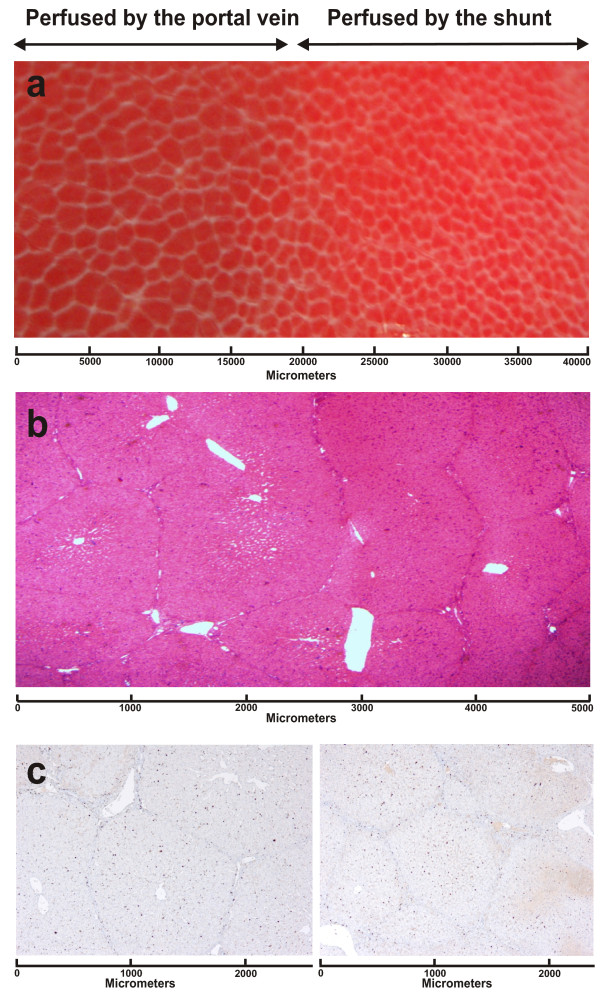
**Macro-and microscopic changes after three weeks of shunting**. a) Close-up photograph of the transition zone between shunted and portally perfused in-vivo liver after three weeks. The shunted side exhibits smaller condensed lobuli and a brighter (hyperoxygenized) color, while the portally perfused side exhibits larger lobuli, b) HE stained section of the transition zone showing more condensed lobuli on the shunted side and larger lobuli with dilated portal venules and central veins on the portally perfused side, c) sections from areas perfused by the portal vein and by the shunt showing an even distribution of Ki67 positive cells (control sections of sham and baseline livers all show a lower density of Ki67 positive cells).

Macroscopically, a sharp line of demarcation between the shunted and portally perfused sides of the liver was seen on the organ surface (in vivo) upon relaparatomy at t = three weeks (Fig. 3a). This line corresponded to the transitional zone between segments IV (perfused by the shunt) and V/VIII (perfused by the portal vein). Furthermore, we observed that the liver lobuli had become larger on the portally perfused side.

### Microscopic changes

On microscopic examination with HE staining (of biopsies taken from the chronic experiments), the lobuli on the shunted arterialized side exhibited condensed, smaller liver lobuli. However, reticulin staining revealed no increase in connective tissue deposition between portal triads. Furthermore, no apparent bile duct hyperplasia could be seen or overt signs of damage due to hyperperfusion. On the portally perfused side, the lobuli were expanded, the hepatocytes larger (increased cytoplasm), and the sinusoids, portal venules as well as the central veins were dilated. There were no differences in the density of Ki67 positive cells or Phosphohistone H3 positive cells between the two sides (Fig. 3b, c). Control sections from sham animals and at baseline before shunting revealed uniformly less Ki67 positive cells in the liver lobuli, tentatively reflecting the pre-interventional normal state.

### Biochemical/cytokine analyses (acute experiments)

There were no statistically significant changes in the concentration of ALAT, ASAT, GT, BIL or ALP at any time nor were there any differences in trends between shunt and sham groups.

Serum IL-1 concentration increased slightly but remained statistically unchanged in the sham experiments. In the shunt experiments, IL-1 concentration reached a peak value (63 ± 93 pmol/l) at t = 4 hours after shunt opening (*p *= 0.009). Serum IL-6 remained unchanged in the sham experiments. In the shunt groups, IL-6 reached a peak value of 596 (± 722 p mol/L) at t = 4 hours (*p *= 0.004). TNF-α was at most time points undetectable in the sham groups. However, in the shunt group we found a peak value of 20 (± 24 pmol/L) at t = 4 hours (*p *= 0.0009). IL-10 concentrations increased in both groups reaching a maximum value of 12 (± 14 pmol/L) in the shunt group (*p *= 0.0007) and 8 (± 9 pmol/L) in the sham group (*p *= 0.004), both at t = 2 hours. There were no significant differences in concentrations of the above cytokines in the venous blood draining the shunted segments and in blood draining the portally perfused segments in the shunted animals - the differences were found between the shunt and sham animals as a whole.

### Gene expression (Additional file 2: Table S2, for full name and synonyms of gene abbreviations used in the following text)

**Figure 4:**
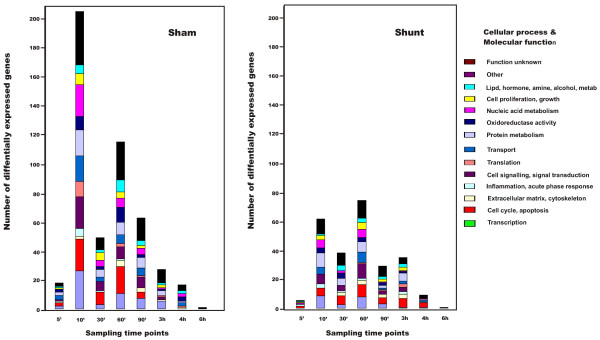
**Functional distribution of differentially expressed genes**. Illustration of differentially expressed genes at given time points sorted by genetic function according to Gene Ontology in the shunted and sham pigs (contrasts within time points).

By analyzing differences between the shunt and sham groups at individual sampling time points and examining potential functions of the gene products by categorization according to cellular process and molecular function (Gene Ontology) we found that in terms of genetic function, although there were many genes whose expression differed in the two groups at each time point of sampling after shunt opening and sham surgery, the functional distribution of the potential gene products were similar in both groups. However, there were far more genes differentially expressed in the sham group (Fig. 4).

By analyzing differential gene expression over time within the sham and shunt groups, we found major quantitative and qualitative differences. Not only were there by far more genes differentially expressed in the sham group, but genes associated with the regulation of the cell cycle and apoptosis found in previous studies [16,18-20] were more prominent (Additional file 3: Table S3).

### Cell cycle/apoptosis genes differentially expressed in the shunt series (Additional file 3: Table S3)

*PTMA *(upregulated at 3h-1' interval) dually regulates apoptosis by modulating the caspase cascade as it inhibits the activation of procaspase 9 by Apaf1 but at the same time, inhibits caspase 9 itself [28]. *SCYL 2 *(downregulated at 3h-1' and upregulated at 6h-1') is associated with *SCYL 1*, a gene involved in centrosome formation and mitosis [29]. *MAPK8IP2*, (downregulated at 6h-1') potentially counteracts apoptosis [30].

### Cell cycle/apoptosis genes differentially expressed in the sham series (Additional file 3: Table S3)

Upregulated genes: *KIF 4A *(5-1') and *KIF 1B *(6h-1') are associated with *KIF 20A*, which regulates the organization of the microtubuli apparatus, involved in cell division [31]. *NME1 *(5-1', 30-1', 3h-1') potentially counteracts DNA damage and cytolysis [32,33]. *MAPK8IP2 *(5-1', 2h-1') inhibits apoptosis [30]. *UBE2C *(5-1', 2h-1', 4h-1') facilitates progression of the cell cycle via *APC *activation and increased cyclin A [34]. *UBE2M *(hUbc12) is a conjugating enzyme for NEDD8, involved in the ubiquitinylation of cell-cycle factors involved in the G1/S transition [35]. *IGFBP3 *(5-1') is associated with *IGFBP5*, which in turn may lead to cell cycle arrest in the G2/M phase [36]. *CDK5 *(10-1') associated with *CDK6 *promotes cell cycle transition in the G1 phase [37].

Downregulated genes: *MAPK13 *(5-1', 30-1', 3h-1', 4h-1', 6h-1') is one of several protein kinases activated by cellular stresses (including oxidative stress) and cytokines IL-1 and TNFα and has been found to be a downstream carrier of the PKCdelta-dependent death signal [38]. Over expression of *BTG3 *(10-1', 4h-1') has been shown to impair serum-induced cell cycle progression from the G0/G1 to S phase [39]. *UBE2C *promotes progression of the cell cycle [34]. *Bcl-rambo *(2h-1', 3h-1', 4h-1') is a *Bcl-2 *member that induces cell death [40]. *MAPK6 *(3h-1', 6h-1') - over expression of this gene in *NIH 3T3 *cells has been seen to inhibit DNA synthesis and G1 phase arrest [41] and the nucleocytoplasmic shuttling of *ERK3 *regulates its inhibitory action on cell cycle progression [42]. *MDM2 *transcriptional (3h-1', 6h-1') products form complexes with p53 in the G0/G1 phases of the cell cycle and inhibit the G1 arrest and inhibitory functions of p-53 [43].

## Discussion

In this study we find that an isolated increase in sinusoidal flow does not have the same macroscopic, microscopic or genetic impact on the liver as that seen in the liver remnant after partial hepatectomy. Our findings indicate that increased sinusoidal flow may not be a sufficient stimulus in itself for the initiation of liver regeneration.

On histological examination of the transition zone between the shunted and portally perfused sides (Fig. 3), we found the liver lobuli larger on the portally perfused side as previously observed by other investigators [44]. The expansion was the result of not only slightly congested sinusoids, but also by, in general, larger hepatocytes. These changes suggests to us that after three weeks of mainly portal perfusion (the right hepatic artery was intact) to segments I, V, VI, VII and VIII, the metabolic and hepatotrophic stimuli from the splanchninc blood results in selective growth of these segments, independently from the shunted contra lateral side (segments II, III and IV). The finding that the proliferative index and phosphohistone H3 distribution is similar in both sides at t = 3 weeks, suggests that this selective growth may be the result of hepatocyte hypertrophy.

Microarray analysis of the liver biopsies (from the acute series) indicate that the shunting had a quantitative impact on gene expression in the shunted segments as compared with the gene expression in the same segments in the sham animals, the effect being a relative general down-regulation in transcriptional activity in the shunted liver (Fig. 4).

On the basis of microarray analysis of biopsies from the shunted liver segments and sham livers we found that not only were there by far more genes differentially expressed in the sham livers, but genes associated with the regulation of the cell cycle and apoptosis found in previous studies [16,18,20,21] were more prominent (Additional file 3: Table S3).

Specific evaluation of the differential expressed genes regulating the cell cycle and apoptosis in the shunt group revealed that they were not only quantitatively insignificant compared to the sham group, but also qualitatively equivocal as their potential functions diverged (some promote and some inhibit mitosis). On the contrary, all upregulated genes associated with the cell cycle and apoptosis in the sham group potentially promote cell division and inhibit apoptosis (with the exception of *IGFBP5*). Furthermore, with the exception of *UBE2C*, the differential expression of all downregulated genes associated with the cell cycle in the sham group also favored cell cycle progression (Additional file 3: Table S3).

As a whole, the microarray analysis of the immediate gene expressional activity in the shunted and sham livers indicate a relative increase in general transcriptional activity and a more pronounced activity of cell cycle promoting genes in the sham livers relative to the shunted livers.

When comparing gene expression during aorto-portal shunting in the present study to the profiles found after liver resection [21] we find two differentially expressed genes, common to both interventions, both involved in apoptosis signalling. *PTMA *was upregulated at 3 hours after a high pressure liver resection and aorto-portal shunting respectively, and *MAPK8IP2 *was upregulated 90 minutes after a high pressure liver resection and after 6 hours of aorto-portal shunting. The differential expression of these genes tentatively reflects the large hemodynamic impact of both interventions on cellular stress and apoptosis mechanisms.

How can we explain our observation that the non-shunted, portally perfused side of the liver grows after three weeks, resulting in the liver's supranormal weight gain to 3.9% of body weight while the weight percentage of the shunted side does not change in the same period? Firstly, the shunted blood was arterialized. It may be that this increase in oxygenation may have been unphysiological to such an extent that any potential growth stimulating flow stimulus on the endothelial surface was suppressed. However, a high oxygen tension in portal venous blood has been shown to be beneficial for regeneration after extended PHx in rats and for the outcome of acute liver failure in swine [45,46]. Furthermore, analysis of the flux of liver enzymes, GT, ALP and bilirubin flux across the liver bed and cytokine analysis of blood draining the shunted segments in the acute series, and histological analysis of HE stained sections, does not suggest any immediate deleterious effect on the liver parenchyma as a result of the shunting.

Secondly, ligating the left portal vein branch proximal to the anastomosed aortoportal shunt results in a portal pressure increased from 6.22 mmHg to 8.55 mmHg (*p *< 0.05) however, the flow per gram liver in these portally perfused (not shunted) segments remained unchanged (1.57 to 1.53 mL/gram/minute, not significant) whereas the flow in the shunted segments increased significantly from an average of 0.61 to 2.89 mL/gram/minute after shunt opening giving a 4.75 fold increase in flow which is similar to the flow increase seen after a 75% PHx [21]. Thus, it may be that it is not the quantity of blood perfusing the liver sinusoids in the remnant which is detrimental to liver regeneration, but rather the quality of the blood (with hepatotrophic factors) as previously suggested by Michalopoulos [47]. Supportive of this theory is the findings of Ladurner et al. where extended hepatic resection with or without decompressive portocaval shunting (and thus significant differences in flow in the liver remnant) did not reveal differences in liver regeneration [48]. Conceivably equally important, are the increased metabolic tasks per gram remaining liver imposed on the liver remnant which may lead to its growth.

We maintain, on the basis of this experiment, that the flow theory of increased shear stress as a primary stimulus to liver regeneration is questionable because it is the non-shunted, portally perfused side which hypertrophies despite the fact that flow per gram liver on this side remains unchanged. In contrast to this, the shunted segments exhibited contracted lobuli, no increase in volume and a general downregulation in transcriptional activity. We suggest that the portally perfused side of the liver hypertrophied due to a combination of increased metabolic demand (due to the functional deficiency of the shunted side) and the presence of hepatotrophic growth factors in the portal perfusate.

Finally, is it justifiable to study the process of liver regeneration without performing a resection? In our opinion, yes, because the moment one performs a liver resection, the relative increase in growth factors supplied, and the increase in metabolic demand on the liver remnant confounds the study of an isolated increase in flow per gram remaining liver parenchyma. It is therefore necessary to create an "unphysiological "state to study an isolated phenomenon in vivo.

## Conclusions

On the basis of the present study we conclude that an isolated acute and chronic increase in sinusoidal flow does not have the same genetic, microscopic or macroscopic impact on the liver as that seen in the liver remnant after partial hepatectomy, indicating that increased sinusoidal flow may not be a sufficient stimulus in itself for the initiation of liver regeneration.

## Competing interests

The authors declare that they have no competing interests.

## Authors' contributions

KEM authored the study protocol, performed all surgical experiments, interpreted all results drafted and revised the manuscript. LNC was responsible for all aspects of the microarray analysis including parts of the biostatical analysis. IN made substantial contributions to data acquisition. PS conducted and supervised the biostatistical analysis of the microarray data. EM was responsible for the preparation, analysis and interpretation of histological sections. CB supervised the microarray analysis and made contributions to its biological interpretation. AR was responsible for conceiving the protocol hypothesis and study design and supervised manuscript drafting and revising its intellectual content.

All authors have read and approved the final manuscript.

## Supplementary Material

Additional file 1 **Tabular data 1**. Hemodynamics and liver weight changes in acute- and chronic series. Click here for file

Additional file 2 **Tabular data 2**. Full name and synonyms of gene abbreviations used in the article text. Click here for file

Additional file 3 **Tabular data 3**. Differentially expressed genes regulating cell cycle and apoptosis. Light grey correspond to upregulated genes and dark grey highlights the downregulated ones. Click here for file
