# Supplementary material for: Increased sinusoidal flow is not the primary stimulus to liver regeneration
Source: Comp Hepatol. 2010 Jan 20;9:2. doi: 10.1186/1476-5926-9-2 (PMC2819042; doi:10.1186/1476-5926-9-2)
Supplement: Additional file 1 — Tabular data 1. Hemodynamics and liver weight changes in acute- and chronic series. [file 1476-5926-9-2-S1.PDF]

Shunt acute series

	Units of Measurement	Before Shunt Opening	After Shunt Opening	p - value
Mean Arterial Pressure	mmHg	90.3	70.3	0.01
Systemic Vascular Resistance	mmHg/min/mL	16.5	11.2	0.002
Heart Rate	Beats/minute	100	150	<0.05*
Cardiac Output	mL/minute	5.01	6.65	ns
Flow to segments II, III, IV	mL/minute	221	1050	0.001
Flow / gram liver to segments II, III, IV	mL/gram/minute	0.61	2.89	0.001
Flow to segments I, V, VI, VII, VIII	mL/minute	647	636	0.01
Flow / gram liver to segments I, V, VI, VII, VIII	mL/gram/minute	1.57	1.53	ns
Portal Venous Pressure	mmHg	6.22	8.55	<0.05
Flow in the left hepatic artery	mL/minute	169	122	0.023
Flow in the right hepatic artery	mL/minute	85	46	0.022
Free Left Hepatic Venous Pressure	mmHg	3.3	3.7	ns
Free Right hepatic venous Pressure	mmHg	3.3	3.1	ns
Wedged Left Hepatic venous pressure	mmHg	2.3	8	0.003

Sham acute series

	Units of Measurement	Before Sham Surgery	After Sham Surgery	p - value
Mean Arterial Pressure	mmHg	88.5	85.5	ns
Systemic Vascular Resistance	mmHg/min/mL	17.7	15.9	ns
Heart Rate	Beats/minute	98	95	ns
Cardiac Output	mL/minute	4.26	4.22	ns
Flow to segments II, III, IV	mL/minute	207	-	-
Flow / gram liver to segments II, III, IV	mL/gram/minute	0.637	-	-
Flow to segments I, V, VI, VII, VIII	mL/minute	652	-	-
Flow / gram liver to segments I, V, VI, VII, VIII	mL/gram/minute	1.7	-	-
Portal Venous Pressure	mmHg	6	5	ns
Flow in the left hepatic artery	mL/minute	142	122	ns
Flow in the right hepatic artery	mL/minute	55	48	ns
Free Left Hepatic Venous Pressure	mmHg	2.5	2.1	ns
Free Right hepatic venous Pressure	mmHg	2.3	2.7	ns
Wedged Left Hepatic venous pressure	mmHg	4.8	5.4	ns

Shunt chronic series

	Units of Measurement	t = 0	t = 3 weeks	p - value
Shunt flow to segments II, III, IV	mL/minute	1007	1496	0.004
Weight of segments II, II, IV	grams	314	633	0.0001
Flow / gram liver in segments II, III, IV	mL/gram/minute	2.97	2.38	0.045
Portal flow to segments I, V, VI, VII, VIII	mL/minute	459	1120	0.008
Weight of segments I, V, VI, VII, VIII	grams	412	1034	0.0001
Flow / gram liver in segm. I, V, VI, VII, VIII	mL/gram/minute	2.07	1.08	0.0001
Body weight	grams	27600	41800	0.0001
Liver weight	grams	754	1667	0.0001
Ratio of liver to body weight	%	2.74	3.99	0.004
Weight of segments II, II, IV	grams	341	633	0.001
Ratio of segm. II, II, IV to body weight	%	1.24	1.52	ns
Weight of segments I, V, VI, VII, VIII	grams	412	1034	0.0001
Ratio of segm. I, V, VI, VII, VIII to body wt	%	1.49	2.47	0.002
