# Supplementary material for: Increased sinusoidal flow is not the primary stimulus to liver regeneration
Source: Comp Hepatol. 2010 Jan 20;9:2. doi: 10.1186/1476-5926-9-2 (PMC2819042; doi:10.1186/1476-5926-9-2)
Supplement: Additional file 2 — Tabular data 2. Full name and synonyms of gene abbreviations used in the article text. [file 1476-5926-9-2-S2.PDF]

Gene abbreviation	Gene name / synonyme
Apaf 1	Apoptotic protease activating factor 1
APC	Adenomatous polyposis coli
Bcl-rambo	B-cell CLL/lymphoma rambo
BTG3	B-cell translocation gene
CDK5	Cyclin dependent kinase 5
CDK6	Cyclin dependent kinase 6
ERK3	Mitogen-activated protein kinase 12
IGFBP3	Insulin-like growth factor-binding protein 3
IGFBP5	Insulin-like growth factor-binding protein 5
KIF 20A	Kinesein family member 20 A
KIF 4A	Kinesein family 4A
KIF B	Kinesein family member B
MAPK13	Mitogen activated protein kinase 13
MAPK6	Mitogen activated protein kinase 6
MAPK8IP2	Mitogen activated protein kinase 8-interacting protein 2
MDM2	Mouse double minute 2 homolog
NEDD8	Neural precursor cell expressed, development downregulated 8
NFkB	Nuclear factor kappa B
NME1	Nonmetastatic cells 1, protein expressed in
PTMA	Prothymosin, alpha
SCYL 2	Telomerase associated protein
UBE2C	Ubiquitin-conjugating enzyme E2C
UBE2M	Ubiquitin-conjugating enzyme 2M
