# Supplementary material for: Increased sinusoidal flow is not the primary stimulus to liver regeneration
Source: Comp Hepatol. 2010 Jan 20;9:2. doi: 10.1186/1476-5926-9-2 (PMC2819042; doi:10.1186/1476-5926-9-2)
Supplement: Additional file 3 — Tabular data 3. Differentially expressed genes regulating cell cycle and apoptosis. Light grey correspond to upregulated genes and dark grey highlights the downregulated ones. [file 1476-5926-9-2-S3.PDF]

	Within group contrasts for time periods							
	**5-1'**	**10-1'**	**30-1'**	**90-1'**	**2h-1'**	**3h-1'**	**4h-1'**	**6-1'**
**Shunt**	0	0	0	0	0	PTMA<br>SCYL 2		SCYL 2<br>MAPK8IP2
**Sham**	KIF 4A<br>NME 1<br>MAPK8IP2<br>UBE2C<br>UBE2M<br>IGFBP3<br>MAPK13	CDK 5<br>UBE2M<br>UBE2JI<br>BTG3	NME1<br>MAPK13<br>UBE2C<br>UBE2JI	UBE2JI	MAPK8IP2<br>UBE2C<br>Bcl-rambo	NME1<br>Bcl-rambo<br>MAPK13<br>MAPK6<br>MDM2	UBE2C<br>Bcl-rambo<br>MAPK13<br>BTG3	KIF1B<br>SCYL 2<br>MAPK6<br>MAPK13<br>MDM2
